# Theranostics approach in drug development: is there study efficiency when the prevalence of the molecular target is very high?

**DOI:** 10.7150/thno.71788

**Published:** 2022-03-28

**Authors:** Sue-Jane Wang, Anthony Fotenos, Shane C. Masters, Louis Marzella

**Affiliations:** 1 Division of Biometrics I, Office of Biostatistics, Office of Translational Sciences, Center for Drug Evaluation and Research, U.S. Food and Drug Administration.; 2 Division of Imaging and Radiation Medicine, Office of Specialty Medicine, Office of New Drugs, Center for Drug Evaluation and Research, U.S. Food and Drug Administration.

## Background

Diagnosis and therapy share a nuanced relationship in medicine. While most patients experience diagnosis prior to therapy in temporal sequence, causal linkages between diagnosis and therapy are generally indirect because most sources of diagnostic information are “stand alone”: designed to update understanding of anatomy, function, and/or disease in a patient 1. In turn, updated understanding, ordinarily based on integrating multiple sources of diagnostic information and expert knowledge, serves to guide therapy.

Against the background of this typical (indirect) approach, multiple new approaches have been evolving in recent years in response to innovations in precision medicine. These approaches share a defining characteristic: more direct linkage by design between diagnosis and therapy. In general, for direct approaches, the distinction between “positive” (or “present”) and “negative” (or “absent”) diagnostic information is specifically designed to guide decisions between at least two potential actions at the patient level and limited in scope with respect to a particular therapy (or to a narrowly defined group of therapies).

The distinction between indirect and direct linkages between diagnosis and therapy also applies to medical product regulation. Examples of regulatory frameworks that feature more direct linkages include co-development, companion diagnostics, trial design enrichment, and theranostics 2. Despite considerable conceptual overlap, these frameworks have somewhat distinct points of emphasis. Namely, the trial enrichment framework has evolved primarily to increase efficiency at the trial design stage of a single medical product development 3. The framework of companion diagnostics has evolved within a context in which the source of diagnostic information is typically a to-be-(co)marketed *in vitro* diagnostic device 4, e.g., pembrolizumab in patients with metastatic non-small cell lung cancer whose tumors express PD-L1 as determined by an FDA-approved test 5, trastuzumab and hyaluronidase-oysk in patients with breast cancer whose tumor over express HER2 based on an FDA-approved test 6, etc.

More recently, the framework of theranostics (a combination of the terms “Therapeutics and Diagnostics”) is evolving to address the growth of new radiopharmaceuticals with shared mechanisms of action for imaging and therapy 2,7. Approximately 90% of prostate cancers overexpress PSMA (Prostate Specific Membrane Antigen), a growing molecular target of both diagnostic and therapeutic radiopharmaceutical development 8-11. Because of the high prevalence of PSMA in patients with prostate cancer, it is of importance to better understand the clinical utility of theranostic approaches that combine PSMA-based radionuclide imaging and prostate cancer therapy for co-development consideration.

## Clinical trials for theranostics development

For a theranostic development where patient selection is to be based on molecular targeting using a diagnostic radiopharmaceutical, early phase trials may pursue a targeted approach only for the therapeutic radiopharmaceutical. As a result, it may not be well-understood if a targeted approach based on a diagnostic radiopharmaceutical is truly beneficial without formally assessing treatment effect in the off-target patient population.

When the molecular target is in truth predictive of treatment effect, we may consider the setting where there is null (or no) treatment effect in patients without the molecular target. The study design efficiency using a theranostic approach to select patients and only investigate the treatment effect in patients with the molecular target can be substantial if the molecular target is known to predict treatment effect. In this scenario, a theranostic approach can avoid exposure of off-target patients to an “ineffective” treatment. However, often 'known to predict treatment effect' is founded on a mechanistic hypothesis.

Treatment effect is generally defined as the absolute difference or the relative effect measured by the primary efficacy endpoint between the treated arm and the control arm in a two-arm randomized, controlled clinical trial. Below, we illustrate the potential advantages of a theranostic approach in terms of sample size saving depending on whether the characteristics of a molecular target are prognostic, predictive, or prognostic-predictive of the disease and/or treatment effect that is under investigation for drug development.

Let Δ be the effect size parameter for all patients satisfying inclusion/exclusion criteria, n, studied with a non-targeted approach. The non-targeted approach includes, Δ+, the effect size for targeted patients with sample size n+, and Δ- , the effect size for off-target patients with sample size n-. Here, we assume equal sample size between the treatment and the control in a two-arm randomized controlled trial.

## Predictive of treatment effect

If no treatment effect exists in the off-target patient population, Δ- = 0, the off-target patients included in the all-patients study contribute 'null' treatment effect and the sample size of the off-target patient population can be saved if a targeted approach is employed. Mathematically, this is equivalent to calculating the percentage of the sample size saved using a targeted approach. This percentage through sample size planning formulas for non-targeted and targeted designs with the same statistical power can be simplified to (1).

Percentage of sample size saving = (n - n+) / n

= 1 - (Δ/Δ+)^2^ (1)

**Figure 1:**
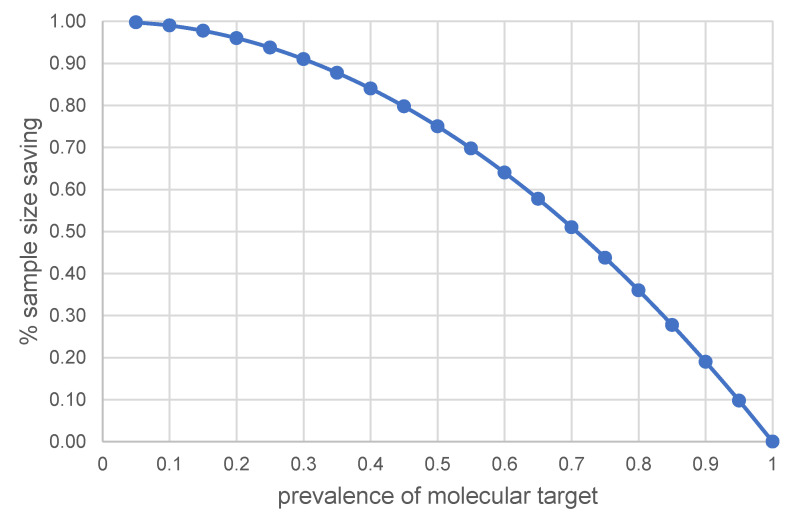
The % of sample size saving when molecular target is predictive of treatment effect

Figure 1 presents the percentage of sample size saving with a targeted approach as a function of the prevalence of molecular target in the diseased population due to a predictive treatment effect. For instance, the sample size saving could be approximately 75% when the prevalence of molecular target is 50%. The issue at stake is whether sample size saving is worthwhile using a targeted approach when the prevalence of molecular target in the diseased population is high. From Figure 1, the sample size saving (or event number saving for a time to event endpoint by applying the algorithm in 12) is approximately 36% (or 43%) with 80% prevalence, approximately 28% (or 34%) with 85% prevalence, approximately 19% (or 24%) with 90% prevalence and approximately 10% (or 13%) with 95% prevalence. The trend shown in Figure 1 generally holds for a single primary efficacy endpoint.

## Prognostic of disease or disease outcome

When a molecularly targeted therapeutic is used, the corresponding diagnostic agent may not always be predictive of treatment effect. This might be due to incomplete understanding of the mechanism of the therapeutic or limitations of the diagnostic test. However, even though the hypothesized predictive molecular targeting may not be the true state of nature, molecular targeting of patients' baseline characteristics or baseline biomarkers may still provide utility for prognostic enrichment in an adequate and well-controlled clinical trial 3.

When serving as a prognostic factor, a molecular target could be used to select patients with a greater likelihood of having a disease-related endpoint event or a substantial worsening in disease condition. This concept is separate from a comparison of two treatment groups for treatment effect assessment in a randomized clinical trial. Prognostic enrichment can increase the study power resulting in a higher probability of identifying a favorable treatment effect, if it exists, compared to studying unselected patients without employing an enrichment strategy.

**Figure 2:**
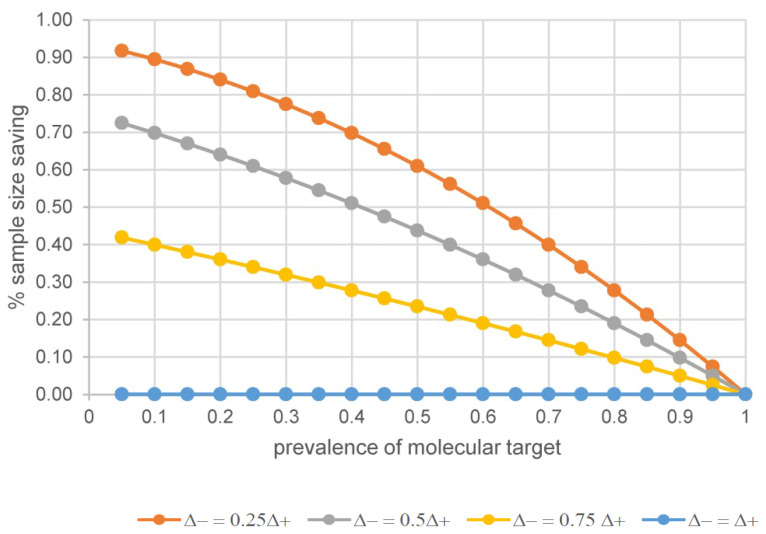
The % of sample size saving when molecular target is prognostic of disease and/or predictive of treatment effect.

It is important to recognize that for a baseline prognostic factor, the relative treatment effect is expected to be the same in the target selected patients and the off-target patients 12,13. Strictly speaking, there is no sample size saving with prognostic enrichment if all patients (target negative patients and target positive patients) are studied and relative treatment effect is of main interest; see the flat curve for Δ- = Δ+ in Figure 2. Studies using prognostic enrichment as a selection or study entrance criteria have been accepted as a basis of drug approval for marketing without a requirement to study broader populations 12. It is also important to assess the benefit/risk balance of an experimental treatment between high-risk and low-risk patients when absolute risk difference is the primary measure of treatment effect.

## Prognostic-Predictive of both disease and treatment effect

A molecular target may possess the characteristic of being both prognostic of disease state and predictive of differential treatment effects between molecular target positive patients and off-target patients, as discussed in 13. In other words, Δ+ > Δ- > 0. When a null effect cannot be assumed in the off-target patients, the clinical utility of the molecular target could be considered prognostic-predictive of treatment effect. To demonstrate this prognostic-predictive relationship, one needs a comparative randomized trial that enroll patients who are positive for the target, e.g., over expression of PSMA, and patients who lack the target, i.e., off-target patients.

The de-enriched off-target patients might take a longer time to experience disease-related endpoint events. As a prognostic-predictive molecular target, when incorporated in a randomized controlled trial, the magnitude of the relative treatment effect or absolute treatment effect in the off-target patients could be smaller. The clinical trial will likely have a longer duration and require a larger sample size if off-target patients are included for investigation.

Let the effect size in the off-target patient set, Δ-, be *p* fraction of the effect size in the targeted patient set. That is, Δ- = *p* * Δ+. In addition, let *f* be the prevalence of the molecular target in the intended to treat patient set. The sample size saving using a theranostic approach for a prognostic-predictive molecular target can be formulated, see (2).

Percentage of sample size saving = 1 - {(*f* + (1-*f*) * *p*}^2^ (2)

The top three curves in Figure 2 illustrate that compared to a non-targeted design with equal power, the percentage of sample size saving with a targeted design increases as the true treatment effect in the off-target patients relative to that in the target positive patients decreases from 75%, to 50%, to 25%. Using 50% prevalence as an example, the percentages of sample size saving are 23%, 44% and 61% with the off-target effect size being 75%, 50%, 25% of the targeted effect size, viz., *p* = 0.75, 0.5, 0.25, respectively. The percentage of sample size saving becomes 5%, 10% and 14% when the prevalence of molecular target is high, around 90%.

## Discussion

We use the PSMA example to discuss the issue of high prevalence with a theranostic approach. Sweat et al 9 and Paschalis et al. 14 have reported that metastatic lesions are PSMA positive in most patients with metastatic castration-resistant prostate cancer (mCRPC). High PSMA expression has been shown to be an independent biomarker of poor prognosis across anatomical sites and throughout most of the course of prostate cancer 8,15,16. High expression of PSMA has also been shown to be independently associated with reduced survival 17. It has also been reported that patients with mCRPC and low PSMA expression have poor prognosis and short survival 18. This effect might in part reflect neuroendocrine differentiation of prostate cancer lesions, which is associated with low PSMA expression 19.

PSMA has been identified as a promising target for molecular imaging of prostate cancer and for targeted radionuclide therapy of patients with mCRPC 20,21. The PSMA-targeted PET radiopharmaceuticals not only have been found to have diagnostic accuracy for visualizing sites of prostate cancer, but also paved the way for a theranostic approach 22. Here, from a trial enrichment perspective, the theranostic principle overlaps with the concept of a predictive biomarker (e.g., sufficient uptake on a PSMA-targeted PET scan in putative sites of disease), followed by an individualized treatment with a therapeutic agent, e.g., by using ^177^Lu-labeled, PSMA- targeting radiopharmaceuticals 23,24.

With 90% prevalence of PSMA expression 8-11,25, sample size saving could be approximately 10% to 13% if PSMA positivity is truly predictive of treatment effect in therapeutics trials. From a clinical trial perspective, if the cost of screening PSMA positive patients is less than studying 10% to 13% more patients, one can consider the theranostic strategy to be cost-effective. In addition to cost, it is worthwhile noting that radionuclide therapeutic treatment may require high levels of tremendous radiation exposure to normal organs, e.g., 177Lu-PSMA; it is recommended that such safety concerns be considered in a theranostic strategy.

The key question, however, is what should be a reasonable sample size for studying off-target patients? Or, should molecularly off-target patients be studied at all?

In general, determining the need to characterize the treatment effect in molecular off-target patients should be based on potential benefits and risks in that patient population. FDA enrichment strategy guidance 3 provides two key considerations for possible support of less or sometimes no collection of information on the enrichment-factor-negative population:

- A clear pathophysiologic basis for concluding that the nonenriched population will not respond.
- Early clinical studies that show very marked differences in response between the enrichment and non-enrichment populations.

In the case of molecular targeting, the first bullet above corresponds to patients lacking the molecular target of the drug. FDA enrichment strategy guidance 3 acknowledges that in such cases, it may be acceptable to provide support using nonclinical or clinical pharmacology and biomarker studies. In some cases, such information could be obtained from the literature.

A central issue with imaging radiopharmaceuticals is that there are multiple sources of uncertainty, and the impact of the uncertainties associated with the linked diagnostic and the therapeutic should be considered in clinical trial designs for theranostics drug development. It is recognized that usually a pre-specified threshold evaluated by human readers is relied upon in classifying patients into presence or absence of the molecular target of interest 26. In other words, uncertainty in classifying a patient having the particular molecular target remains to be characterized or understood. If uncertainty is associated with either reader variability or threshold setting, then those uncertainties should be accounted for in the trial planning whenever possible. Otherwise, the observed efficacy evidence would need to be carefully interpreted in view of reader variability or uncertainty in threshold setting. In principle, if the threshold is used to select patient for targeted enrichment, the threshold needs justification including validation of a cutoff threshold for measurement reliability. Pre-specification of the cutoff threshold alone without justification would be less optimal in confirmatory clinical trials.

The greater the uncertainty regarding the cutoff threshold to establish the targeted mechanism for patient selection and/or the greater the responsiveness of off-target patients if preliminary data exists, the more advisable it would be to include a reasonable sample size of off-target patients, perhaps using an adaptive design to first include all patients and then exclude such patients if they are found to be not responding based on pre-specified criteria or experiencing more serious adverse reactions in an ongoing trial 27; the off-target patients prior to adaptation would constitute the sample size of the off-target patients. Or, an adaptive design may be pursued to explore the optimal cutoff threshold for theranostic consideration.

A molecularly targeted approach serving as prognostic and/or predictive enrichment ensures treatment effect in the selected patients if treatment effect is demonstrated in the selected patients set, and there is little uncertainty in the molecular targeting methodology for selecting patients. The estimates shown in this paper assume certainty in the classification of patients into possessing versus not possessing the molecular target. While mechanisms of action may be shared in theranostics, the less than perfect sensitivity and specificity in patient classification for patient selection in the screening phase can affect the responsiveness to therapy in the treatment phase of a clinical trial. When diagnostic drug is assumed to perform optimally, the saving in sample size can be considerable even when the prevalence of disease is relatively high, e.g., in the 80% to 90% range.

Reducing the uncertainty with the use of an imaging radiopharmaceutical for classifying patients in a theranostic approach is not only desirable for improved accuracy, but it can also pave a clearer path toward efficient clinical trial design. In other words, the higher the accuracy of patient classification is, the smaller the sample size would be, which gains design efficiency with a theranostic approach. The theoretical trend in sample size saving with a truly predictive molecular target, shown in Figure 1, or a prognostic-predictive molecular target, shown in Figure 2, is generally applicable to time to first event endpoints using the metric “percentage of events saving”, which can then be expanded to the percentage of sample size saving for the desired time to first event endpoint, depending on the algorithm used 12, 28, 29.
